# Specific and complete human genome amplification with improved yield achieved by phi29 DNA polymerase and a novel primer at elevated temperature

**DOI:** 10.1186/1756-0500-2-48

**Published:** 2009-03-24

**Authors:** Osama Alsmadi, Fadi Alkayal, Dorota Monies, Brian F Meyer

**Affiliations:** 1 Genetics Department, Research Center, King Faisal Specialist Hospital and Research Center, Riyadh, Saudi Arabia

## Abstract

**Backgrounds:**

Whole genome amplification (WGA) is a practical solution to eliminate molecular analysis limitations associated with genomic DNA (gDNA) quantity. Different methods have been developed to amplify the whole genome, including primer extension preamplification (PEP), degenerate oligonucleotide primed PCR (DOP-PCR), and multiple displacement amplification (MDA). Each of these methods has its own merits and limitations.

**Findings:**

Effects of primer length and composition on amplification quality and quantity were explored in this study at two different temperatures (30°C & 40°C). New primer designs combined with elevated amplification temperature has significantly improved MDA as measured by amplification yield, genome coverage, and allele drop out (ADO) analysis. A remarkable finding was the comprehensive amplification, at 30°C & 40°C, of the human whole genome via the use of GGGCAGGA*N*G hotspot recombination consensus primer. Amplification was characterized by Affymetrix 10K SNP chip analysis. Finally, the use of new primer designs has suppressed the template-independent DNA amplification (TIDA) both at 30°C and 40°C.

**Conclusion:**

The use of new primers in this study combined with elevated incubation temperatures in MDA has remarkably improved the specificity, amplification yield, and suppressed TIDA.

## Background

Whole genome amplification (WGA) is an in vitro method that is used to amplify a genomic DNA (gDNA) sample, and generate amplified DNA for further molecular genetic analyses. WGA is a useful method for production of sufficient DNA quantity from samples with limited DNA content. Several methods have been developed to amplify the whole genome including primer extension preamplification (PEP) [1], and degenerate oligonucleotide primed PCR (DOP-PCR) [2]. Amplification yield and imbalanced amplification in addition to Allele dropout (ADO) associated with these technologies have limited their broad utilization. The most recent advancement of WGA technology was the introduction of multiple displacement amplification (MDA). MDA, unlike PEP and DOP which require PCR cycling for amplification, is an isothermal method that utilizes bacteriophage phi29 DNA polymerase [3] and a random hexamer (NNNNNN) for amplification [4]. MDA has several advantages over other methods mainly due to high processivity of phi29 DNA polymerase which is capable of generating large fragments (>10 kb in size) making it a suitable method for haplotyping, in addition to its proofreading activity which results in much lower misincorporation rates relative to *Taq *DNA polymerase [5-7]. MDA has been broadly used for a wide range of clinical samples. Genome coverage in the amplified DNA from as little as 0.3 ng of template DNA (~45 cells) was equivalent to genomic DNA as assessed by single nucleotide polymorphism (SNP) analysis, and the amplification bias was superior to PEP and DOP [8]. A major limitation of MDA is its utilization for unusual templates such as degraded DNA, or DNA derived from fixed paraffin tissues; cross linked DNA and/or short DNA fragments which are not well tolerated [9]. In an assessment of whole genome amplification methodologies comparing DOP, PEP, Repli-g, and GenomiPhi, all methods induced bias relative to the unamplified DNA, however MDA generated the least bias [10]. From among the different WGA techniques, MDA has been recognized to be the most effective WGA at the present time [11-13]. However, MDA is still facing some challenges such as amplification yield, genome coverage, Template independent DNA synthesis TIDA, and allele drop out (ADO) [14,15]. TIDA has been addressed by technical modifications on the MDA protocol, for examples reducing the reaction volume to nanoliteres resulted in suppression to TIDA [16,17]. Perhaps, the minimal volume in these reactions stoichiometrically favors the primers annealing to their intended template instead of the harmful primer-primer collisions; initiation and spread of TIDA will be constrained in this homogenous microenvironment.

Random primers when used possess the ability to prime on templates that are as complex as the human genome. The number of individual primers of a specific sequence in random primer mixture can be computed from the formula 4^n^, where n is the number of bases incorporated into this primer. A random hexamer (NNNNNN) for instance is constituted of a pool of 4096 (4^6^) primers. The likelihood of a given primer from this primer pool to encounter its annealing site increases as the template complexity itself increases. Therefore, the overall outcome will be a reflection of an oligo-oligo interaction whether being between primers or between primers and template. In the end, quality and quantity are the two most important components which matter in WGA. For these reasons, we have initiated this study to explore the effect of length and composition of different primers on MDA. Variables such as temperature, amplification yield, locus representation, coverage, and allele drop out and bias were revisited.

## Findings

### Methodology

#### WGA primers

A total of 5 primers that varied in sequence and length were used in this study. The primers were out sourced from Metabion (Metabion International AG, Martinsried, Germany). Primers were either non degenerate (consist of one specific sequence), or partially to complete random with their length ranging from 6 to 10 base pairs. The primers were synthesized with phosphothioate (PTO) bonds on their 3' end to protect them from the 3' exonuclease activity of Phi29. NNNN*N*N random hexamer primer utilized by the Repli-g kit was used as reference (Qiagen, Valencia, CA, USA). The remaining 4 primers are as follow: NNNNN*N*N, GGNN*N*N, AGGG*A*G, and GGGCAGGA*N*G (asterisks indicate the position of PTO bonds). These last 4 primers were designed as such to: add a seventh random base relative to the reference hexamer primer (NNNNN*N*N), introduce a 5' GC clamp combined with 4 random bases (GGNN*N*N), utilize a non-degenerate GC-rich primer with high Tm (AGGG*A*G), and a human genome-derived hotspot recombination consensus (GGGCAGGA*N*G).

#### Multiple Displacement Amplification (MDA)

MDA was performed as described previously [18], with some modifications. Four customized 4× amplification mixes were prepared so that each included a unique primer different from that of the commercial Repli-g kit (NNNN*N*N). 50 μL amplification reactions were prepared, and each contained 1× reaction buffer, 50 ng of human genomic DNA (Promega Corporation, Madison, WI, USA), 40 units of ϕ29 DNA polymerase (Repli-g kit, Qiagen). Amplifications were incubated at either 30°C or 40°C for 16 h then terminated by heating to 65°C for 5 min. The DNA concentration of the MDA product was measured using the Picogreen assay (Molecular Probes) according to the manufacturer's instructions.

#### Quantitative PCR (qPCR) Analysis of Amplification Products

Molecular beacon-based qPCR analysis was performed using the ABI 7900 HT sequence detector system according to the manufacturer's specifications (Applied Biosystems). Different molecular beacons specific for 4 genes on 4 different chromosomes were used in these assays. The 4 loci used for locus representation were rs12255372 (Chr10), rs5219 (Chr11), rs1078990 (Chr14), and RAGE (CHr6). Each gene locus was tested in triplicate. Every qPCR reaction consists of 10 μL containing 1× Platinum Taq Polymerase Buffer, 5 mM MgCl2, 1 mM each dNTP, 1 μL ROX Reference Dye (Invitrogen Life Technologies), 1 unit of Platinum Taq Polymerase (Invitrogen Life Technologies), 0.1 μM each of forward and reverse PCR primers, 0.2 μM molecular beacon probe, and 50 μg of MDA-amplified DNA. Human genomic DNA (gDNA; Promega) was used to generate a standard curve of 0, 0.001, 0.01, 0.1, and 1 μg gDNA to quantify the amplified DNA. Loci representation (MDA/gDNA) is reported as the average percentage and is derived as 100 × (loci copy number/microgram of MDA product)/(loci copy number/microgram of gDNA). A value of 100% indicates that the loci copy number for the amplified DNA is equivalent to the loci copy number in the unamplified reference gDNA.

#### Affymetrix SNP chip genotyping

Four different anonymized blood samples derived from one family (parents and two siblings) were amplified either by the Repli-g Kit or by GGGCAGGA*N*G primer. Other primers were not tested. For each sample, unamplified DNA, or amplified DNA using 4 different amplification conditions were analyzed. The amplification conditions were as follow: a) Repli-g kit at 30°C, b) Repli-g at 40°C, c) amplification with GGGCAGGA*N*G at 30°C, and d) amplification with GGGCAGGA*N*G at 40°C. A total of 20 samples (4 unamplified gDNA samples, plus 16 samples representing the different amplification conditions) were assessed by genomewide linkage analysis using the GeneChip Mapping 10K Xba142 SNP Array (Affymetrix). SNP genotypes were obtained by following the Affymetrix protocol for the GeneChip Mapping 10K Xba142 Array [19]. Affymetrix GCOS software (v1.2) was used to obtain raw microarray feature intensities (RAS scores). RAS scores were processed using Affymetrix GDAS (v3.0.2) software to derive the SNP genotypes.

## Results

### MDA with and without gDNA template at 30°C and 40°C

**Figure 1:**
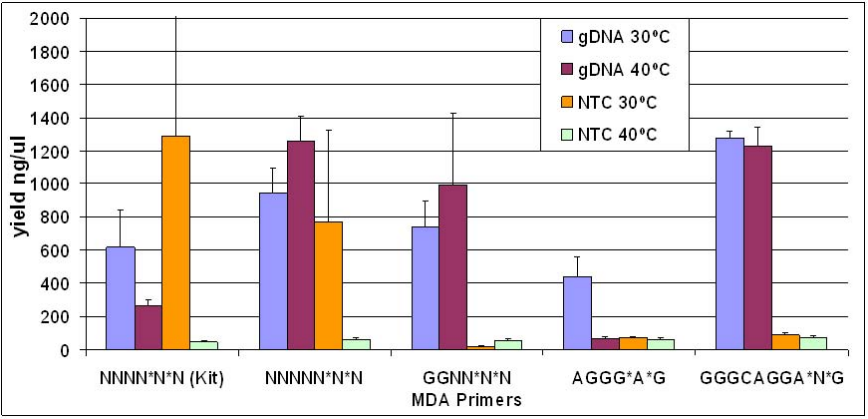
**Comparison of MDA yields in presence and absence of gDNA template**. Four different MDA primers (NNNNN*N*N, GGNN*N*N, AGGG*A*G, and GGGCAGGA*N*G) were used and compared to NNNN*N*N reference primer (used by Repli-g kit). Amplifications were carried out independently at 30°C or 40°C with or without 50 ng of gDNA template in each reaction.

Amplification by Repli-g kit (Qiagen) is routinely carried out at 30°C which is optimal for the activity of phi29 DNA polymerase used in the kit. In an attempt to eliminate or reduce nonspecific template-independent priming, we empirically raised the amplification reaction temperature to 40°C; no other incubation temperatures were explored in these experiments. The amplifications, with and without gDNA template, were compared to the reference incubation temperature (30°C). Various primers that differ in length and sequence were independently used, and subsequently variable amplification product yields were observed and shown in Figure 1.

At 30°C, and in the presence of gDNA template, the MDA yield per each primer was as the following in ng/μl: GGGCAGGA*N*G, 1275; NNNNN*N*N, 947; GGNN*N*N, 740; NNNN*N*N (Kit), 619; AGGG*A*G, 438, and in the absence of template, template-independent DNA amplification (TIDA) yield (in ng/μl) was NNNN*N*N (Kit), 1287; NNNNN*N*N, 770; GGGCAGGA*N*G, 85; AGGG*A*G, 73; GGNN*N*N, 22. When MDA is carried out at 40°C, and in the presence of template, the yield per each primer was as the following in ng/μl: NNNNN*N*N, 1259; GGGCAGGA*N*G, 1228; GGNN*N*N, 993; NNNN*N*N (Kit), 268; AGGG*A*G, 66, and in absence of template, TIDA yield (in ng/μl) was GGGCAGGA*N*G, 72; AGGG*A*G, 64; NNNNN*N*N, 63; GGNN*N*N, 58; NNNN*N*N (Kit), 48.

Relative to the reference primer (NNNN*N*N) and at 30°C, primers GGGCAGGA*N*G, NNNNN*N*N, and GGNN*N*N resulted in an improved template-dependent amplification yield in the order of 2.1, 1.5, and 1.2 folds respectively. AGGG*A*G however resulted in a decreased yield (0.7 fold) relative to the reference primer. At 40°C, the overall yield has improved even further relative to the reference primer; primers NNNNN*N*N, GGGCAGGA*N*G, and GGNN*N*N resulted in 4.7, 4.6, and 3.7 folds increase respectively. AGGG*A*G on the other hand mediated a reduced yield of only 0.2 fold relative to the reference primer. In absence of template, TIDA was dramatically suppressed (48≤TIDA≤72 ng/μl) at 40°C of incubation for all of the primers without exception. The picture was essentially similar at 30°C except for random primers NNNN*N*N (kit) and NNNNN*N*N which mediated significant non-specific amplification yields of 1287 and 770 ng/μl respectively.

### Locus representation analysis

**Figure 2:**
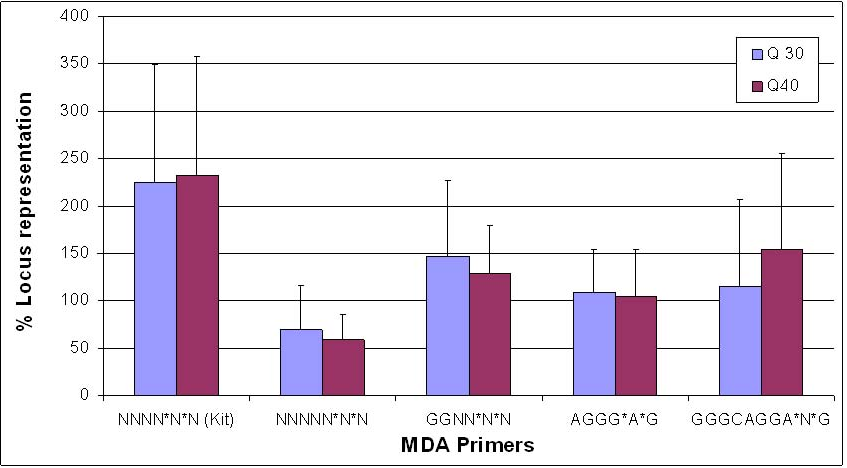
**Locus representation analysis of MDA using quantitative PCR (qPCR)**. Four new MDA primers (NNNNN*N*N, GGNN*N*N, AGGG*A*G and GGGCAGGA*N*G) in addition to the Repli-g kit primer (NNNN*N*N), were utilized in independent amplification reactions to amplify 50 ng of gDNA templates. 100% locus representation indicates complete specificity in the MDA reaction.

Locus representation analysis is an indicator of the specificity of MDA reaction. The ideal average for representation should be 100%. The more loci examined, the closer the average will reflect on the quality of the amplified genetic material. Deviation from the ideal average indicates the presence of non-specific amplification artifacts mainly due to primer dimers. The specificity of the amplification products was characterized by using qPCR assays through the assessment of 4 independent loci that each located on a different chromosome. Real time molecular beacon qPCR assays specific for these loci were performed on the 30°C and 40°C amplified Promega gDNA. Unamplified Promega gDNA was used as reference in these calculations. Locus representation was derived as described in materials and methods, and the average locus representation for the 4 loci is plotted for comparison (Figure 2). Locus representation varied in connection with the utilized primer. Primers NNNN*N*N (Kit), NNNNN*N*N, GGNN*N*N, AGGG*A*G, and GGGCAGGA*N*G when utilized at 30°C resulted in a percent locus representation of 225, 69, 147, 109, and 115 respectively. When these primers were utilized at 40°C, the percent locus representation was 232, 59, 129, 105, and 154 respectively.

### Comprehensive amplification coverage is mediated by GGGCAGGA*N*G primer

Four gDNA samples belonging to one family (F; Father, M; Mother, S; Son, and D; Daughter) were amplified by using GGGCAGGA*N*G primer, and amplification was compared to that accomplished by the reference NNNN*N*N primer (Repli-g kit). Amplification reactions were conducted at 30°C and 40°C resulting in a total of 16 amplification combinations. These and the unamplified gDNA 4 family samples (20 in total) were analyzed by the Affymetrix 10K Xba142 DNA SNP chip. SNP call rate for each of the 20 samples is shown in Table 1. Not a single sample, including the unamplified gDNA, resulted in 100% call rate (i.e. full coverage); call rates were within what we generally see, and there was no specific order (Table 1). The call rates ranged from 92.5% to 97.7% (overall average 96% ± 1.5). The percent average call rates for unamplified gDNA, amplified DNA by Repli-g kit MDA at 30°C and 40°C, and amplified DNA by GGGCAGGA*N*G at 30°C and 40°C were: 95.1 ± 1.6, 96.5 ± 0.94, 96.3 ± 1.1, 96.9 ± 1.4 and 95.0 ± 1.7, respectively. This result clearly suggests no specific trend, in any direction, for any particular sample, primer, or amplification condition.

**Table 1:** Affymetrix 10K Xba142 DNA chip SNP call analysis

Sample	F-G	M-G	S-G	D-G	Average
% SNP Call	93.4	97.2	94.7	95.2	95.1

Sample	F-K30	M-K30	S-K30	D-K30	Average

% SNP Call	96.3	97.7	96.5	95.5	96.5

Sample	F-K40	M-K40	S-K40	D-K40	Average

% SNP Call	97.5	95.1	96.8	95.6	96.3

Sample	F-W30	M-W30	S-W30	D-W30	Average

% SNP Call	97.7	97.7	94.7	97.4	96.9

Sample	F-W40	M-W40	S-W40	D-W40	Average

% SNP Call	95.5	95.8	96.4	92.5	95.0

Percent call rates for gDNA and MDA samples are shown. Samples belong to one family including: Father (F), Mother (M), Son (S), and Daughter (D). G represents unamplified gDNA. K30 and K40 represent MDA reactions using Repli-g kit at 30°C and 40°C, respectively); W30 and W40 represent MDA reactions using GGGCAGGA*N*G primer at 30°C and 40°C, respectively.

### Amplification Allele Drop Out (ADO) assessment

ADO was assessed for the 20 DNA samples described above following Affymetrix SNP genotyping, by performing independently Mendelian concordance check. Normalized Mendelian error check was computed using Affymetrix GeneChip DNA analysis software (GDAS) for this purpose. The 20 samples were grouped into 5 sets where each set contained 4 samples as the following: 1) unamplified gDNA, 2) amplified DNA by Repli-g at 30°C, 3) amplified DNA by Repli-g at 40°C, 4) amplified DNA by GGGCAGGA*N*G at 30°C, and 5) amplified DNA by GGGCAGGA*N*G at 40°C. The percent Normalized Mendelian errors for these 5 sets were 1.45, 1.45, 1.42, 1.32, and 1.64 respectively. The differences in the genotyping errors, as assessed by Chi square analysis, were not statistically significant, indicating that all of the DNA samples (amplified/unamplified) are essentially similar in their quality, and hence the different MDA conditions carried out in these experiments have not introduced bias into the SNP genotyping.

## Discussion

The effects of primer length, composition, and thermal conditions on MDA quantity and quality were revisited through investigating amplification yield, locus representation, coverage, and ADO. It turned out that yield itself is dependent not only on the primer length and composition, but also on the thermal condition. For instance, when amplification is carried out at 30°C, the yields mediated by the three primers GGGCAGGA*N*G, NNNNN*N*N, and GGNN*N*N were increased in 2.1, 1.5 and 1.2 folds respectively relative to the reference primer NNNN*N*N. When amplification was carried out by the same primers at 40°C, the increases in yields were 4.6, 4.7 and 3.7 folds respectively, which reflects even a further improvement on yield. One can relate these significant improvements to the extra length and on-template stability subsequent to these primers' higher Tm relative to the reference primer, and possibly the increased productivity of enzyme at this higher temperature. Additional factors like presence of the GC clamp on 5' end of GGNN*N*N and higher frequency of priming sites in the genome associated with GGGCAGGA*N*G which mimics the mammalian hot spot recombination consensus may also be responsible for these enhanced yields. For applications that demand substantial quantities of DNA like gene mapping and DNA microarrays, the use of these primers for amplification may be desirable when limited template is available as the case for archived genomic materials and paraffin embedded tissues.

AGGG*A*G primer on the other hand gave a relatively reduced yield of 0.7 and 0.2 at 30°C and 40°C respectively. This reduced yield may be seen from a different perspective as positive characteristic, especially when this is considered in the contest of locus representation. Percent locus representation associated with this primer was 109 and 105 at 30°C and 40°C respectively. This suggests a near unamplified (i.e. gDNA like) quality of DNA is generated from the gDNA template by AGGG*A*G primer, which may be a desirable outcome despite the reduced relative yield associated with this primer. Locus representation was less than optimal (69% and 59% at 30°C & 40°C respectively) for NNNNN*N*N random heptamer, which may discourage the use of this primer in MDA despite its significantly improved yield. The slightly high percent locus representation observed for NNNN*N*N (Kit), GGNN*N*N, and GGGCAGGA*N*G at either 30°C or 40°C may or may not be reproduced if more loci are tested. As reasoned by Dean et al. [3], the increased percent locus representation above 100% is blamed on loss of repetitive sequences such as the centromere and telomere repeats during amplification. These elevations though are nothing near what is seen when amplification is carried via the PCR-based methods where products can contain up to 70% amplification artifacts [3,20]. To be objective, future investigations that include more than 4 loci in the locus representation analysis will provide a better indication regarding the performance of different MDA primers. Indeed, when the reference primer NNNN*N*N was investigated, this was the case [18]. For a comprehensive evaluation, only complete whole genome-based assessment will reflect the true make up of the amplified materials.

The optimum temperature for Phi29 is 30°C, however the experience we have suggests increasing the incubation temperature to 40°C does not compromise the polymerase's enzymatic activity. The data also suggest other temperatures between 30°C and 40°C could be used, an area which is worth pursuing in the future. To this respect, such pursuit should put into account the length and sequence of the primers in question, since in our hands trying 8,9, and 10 bp random primers resulted in inefficient and non-specific amplifications (data not shown). This is consistent with Lage et al. study, where they demonstrated an improved genomic coverage when amplification by Bst polymerase is compared to that of phi29 [21]. The former method being conducted at 50°C in addition to using nitroindole-modified primers may have led to the dramatical TIDA reduction in Bst polymerase amplification, a notion that is highly relevant to our similar findings.

Enzyme stabilization against thermal inactivation has been studied in the past, and the use of some additives like non-ionic detergents (e.g. TRITON X-100 and Tween 20) has been shown to stabilize the activity of DNA polymerases [22]. In the current study, no additives were added to the amplifications at this relatively high temperature, and the reasons for maintaining its activity are not clear. In absence of template, we do not really know whether the lack of TIDA associated with some of the new primers was due to lack of primer dimerization or due to deterioration of the polymerization activity of Phi29 DNA polymerase. The second possibility may be excluded since random hexamer and random heptamer both supported TIDA at 40°C. One could also speculate this TIDA may have resulted within an early amplification window before the enzyme's thermal denaturation; picogreen quantification can reveal this possible kinetics and whether the enzyme half-life is affected or not. TIDA is a variable in MDA and could confuse the technology users. It was suggested in previous reports that, in absence of gDNA template, the large amounts of amplified DNA is due to primer-primer directed DNA synthesis [14,15]. Therefore, MDA yield measured with routine quantification techniques does not necessarily indicate a specific amplification. Negative amplification controls (reactions which lack template) do result in efficient amplifications, however they have failed to direct any locus-specific PCR [15]. Conducting MDA at 40°C could present a possible solution for elimination of TIDA. Amplifying scarce templates from few cells or archived precious samples may benefit from this approach.

Despite the apparent success of MDA, ADO remains as a challenge to the technology. Tzvetkov et al. [23] compared call rates between unamplified DNA and MDA samples generated from 6 ng of DNA, and reported a 90% concordance rate which reached 99% by increasing the amount of template. This same study reported a 7% ADO of one polymorphic allele. When cells are used for MDA reactions, ADO appears to occur at random and disappears as the number of cells is increased (10 to 20) in the amplification reaction [24]. To overcome this problem, the use of different lysing conditions, and further rounds of amplifications from diluted MDA products has been suggested [13,15,25]. In this study, Affymetrix 10K SNP chip analysis resulted in a genome-wide coverage with an overall percent call rate of 96 ± 1.5. gDNA and amplified DNA (at 30°C and 40°C) which is directed by GGGCAGGA*N*G gave call rates that are essentially the same (Table 1). Percent trio normalized Mendelian error was fully concordant in family-based sets of samples grouped as unamplified, or amplified by Repli-g kit or by GGGCAGGA*N*G both at 30°C and 40°C. The combined data point out the consistency across all of the different amplification conditions, and indicate no ADO or bias is introduced by these amplifications when compared to the reference gDNA. This is considered a remarkable observation and indicates the comprehensiveness of these amplifications which result essentially in "gDNA equivalent" products which would be suitable for any subsequent molecular assay.

In summary, the work presented here provides an opportunity to explore further MDA and offers a new insight toward some of the technology's variables. The use of new primer designs combined with elevated thermal conditions have demonstrated simultaneously an improved amplification yield and suppressed the undesired TIDA. The main points from this current investigation is to introduce these new primer designs which present a potential benefit to MDA, and to invite further MDA research to study the effects of primer length and composition on amplification quality and quantity, over a spectrum of thermal incubation conditions.

## Abbreviations

WGA: Whole genome amplification; MDA: Multiple Displacement Amplification; PEP: Primer extension preamplification; DOP-PCR: Degenerate oligonucleotide primed PCR; PTO: Phosphothioate; TIDA: Template-independent DNA amplification; ADO: Allele drop out; GDAS: GeneChip DNA analysis software.

## Competing interests

The authors declare that they have no competing interests.

## Authors' contributions

OA designed the study, conducted the work, and wrote the manuscript. FA assessed in execution of the experiments. DM supported the conduct of the experiments. BFM co-directed the work and edited the manuscript. All authors read and approved the final manuscript.

## References

[B1] Zhang L, Cui X, Schmitt K, Hubert R, Navidi W, Arnheim N (1992). Whole genome amplification from a single cell: implications for genetic analysis. Proc Natl Acad Sci USA.

[B2] Telenius H, Carter NP, Bebb CE, Nordenskjold M, Ponder BA, Tunnacliffe A (1992). Degenerate oligonucleotide-primed PCR: general amplification of target DNA by a single degenerate primer. Genomics.

[B3] Dean FB, Hosono S, Fang L, Wu X, Faruqi AF, Bray-Ward P, Sun Z, Zong Q, Du Y, Du J (2002). Comprehensive human genome amplification using multiple displacement amplification. Proc Natl Acad Sci USA.

[B4] Dean FB, Nelson JR, Giesler TL, Lasken RS (2001). Rapid amplification of plasmid and phage DNA using Phi 29 DNA polymerase and multiply-primed rolling circle amplification. Genome Res.

[B5] Eckert KA, Kunkel TA (1991). DNA polymerase fidelity and the polymerase chain reaction. PCR Methods Appl.

[B6] Esteban JA, Salas M, Blanco L (1993). Fidelity of phi 29 DNA polymerase. Comparison between protein-primed initiation and DNA polymerization. J Biol Chem.

[B7] Lundberg KS, Shoemaker DD, Adams MW, Short JM, Sorge JA, Mathur EJ (1991). High-fidelity amplification using a thermostable DNA polymerase isolated from Pyrococcus furiosus. Gene.

[B8] Lovmar L, Fredriksson M, Liljedahl U, Sigurdsson S, Syvanen AC (2003). Quantitative evaluation by minisequencing and microarrays reveals accurate multiplexed SNP genotyping of whole genome amplified DNA. Nucleic Acids Res.

[B9] Wang G, Brennan C, Rook M, Wolfe JL, Leo C, Chin L, Pan H, Liu WH, Price B, Makrigiorgos GM (2004). Balanced-PCR amplification allows unbiased identification of genomic copy changes in minute cell and tissue samples. Nucleic Acids Res.

[B10] Pinard R, de Winter A, Sarkis GJ, Gerstein MB, Tartaro KR, Plant RN, Egholm M, Rothberg JM, Leamon JH (2006). Assessment of whole genome amplification-induced bias through high-throughput, massively parallel whole genome sequencing. BMC Genomics.

[B11] Lasken RS, Egholm M (2003). Whole genome amplification: abundant supplies of DNA from precious samples or clinical specimens. Trends Biotechnol.

[B12] Lovmar L, Syvanen AC (2006). Multiple displacement amplification to create a long-lasting source of DNA for genetic studies. Hum Mutat.

[B13] Panelli S, Damiani G, Espen L, Micheli G, Sgaramella V (2006). Towards the analysis of the genomes of single cells: further characterisation of the multiple displacement amplification. Gene.

[B14] Le Caignec C, Spits C, Sermon K, De Rycke M, Thienpont B, Debrock S, Staessen C, Moreau Y, Fryns JP, Van Steirteghem A (2006). Single-cell chromosomal imbalances detection by array CGH. Nucleic Acids Res.

[B15] Spits C, Le Caignec C, De Rycke M, Van Haute L, Van Steirteghem A, Liebaers I, Sermon K (2006). Optimization and evaluation of single-cell whole-genome multiple displacement amplification. Hum Mutat.

[B16] Hutchison CA, Smith HO, Pfannkoch C, Venter JC (2005). Cell-free cloning using phi29 DNA polymerase. Proc Natl Acad Sci USA.

[B17] Marcy Y, Ishoey T, Lasken RS, Stockwell TB, Walenz BP, Halpern AL, Beeson KY, Goldberg SM, Quake SR (2007). Nanoliter reactors improve multiple displacement amplification of genomes from single cells. PLoS Genet.

[B18] Hosono S, Faruqi AF, Dean FB, Du Y, Sun Z, Wu X, Du J, Kingsmore SF, Egholm M, Lasken RS (2003). Unbiased whole-genome amplification directly from clinical samples. Genome Res.

[B19] Matsuzaki H, Loi H, Dong S, Tsai YY, Fang J, Law J, Di X, Liu WM, Yang G, Liu G (2004). Parallel genotyping of over 10,000 SNPs using a one-primer assay on a high-density oligonucleotide array. Genome Res.

[B20] Cheung VG, Nelson SF (1996). Whole genome amplification using a degenerate oligonucleotide primer allows hundreds of genotypes to be performed on less than one nanogram of genomic DNA. Proc Natl Acad Sci USA.

[B21] Lage JM, Leamon JH, Pejovic T, Hamann S, Lacey M, Dillon D, Segraves R, Vossbrinck B, Gonzalez A, Pinkel D (2003). Whole genome analysis of genetic alterations in small DNA samples using hyperbranched strand displacement amplification and array-CGH. Genome Res.

[B22] Wu AM, Cetta A (1975). On the stimulation of viral DNA polymerase activity by nonionic detergent. Biochemistry.

[B23] Tzvetkov MV, Becker C, Kulle B, Nurnberg P, Brockmoller J, Wojnowski L (2005). Genome-wide single-nucleotide polymorphism arrays demonstrate high fidelity of multiple displacement-based whole-genome amplification. Electrophoresis.

[B24] Handyside AH, Robinson MD, Simpson RJ, Omar MB, Shaw MA, Grudzinskas JG, Rutherford A (2004). Isothermal whole genome amplification from single and small numbers of cells: a new era for preimplantation genetic diagnosis of inherited disease. Mol Hum Reprod.

[B25] Jiang Z, Zhang X, Deka R, Jin L (2005). Genome amplification of single sperm using multiple displacement amplification. Nucleic Acids Res.

